# Beyond surface acting: a mixed-methods investigation of an ACT-based intervention for promoting psychological flexibility and regulatory shift in hotel frontline emotional labor

**DOI:** 10.3389/fpsyt.2026.1785171

**Published:** 2026-05-25

**Authors:** Minnicha Yarosake, Juthamas Haenjohn, Warakorn Supwirapakorn

**Affiliations:** 1 Brain, Mind and Learning Program, Department of Research and Applied Psychology, Faculty of Education, Burapha University, Chonburi, Thailand; 2 Department of Research and Applied Psychology, Faculty of Education, Burapha University, Chonburi, Thailand

**Keywords:** acceptance and commitment therapy, deep acting, emotional acceptance, emotional labor, frontline hotel employees, mixed-methods, psychological flexibility, surface acting

## Abstract

**Background:**

Frontline hotel employees in Thailand’s Eastern Economic Corridor (EEC) routinely suppress authentic emotions to meet organizational display rules—a process known as surface acting—associated with burnout, emotional exhaustion, and diminished well-being. Acceptance and Commitment Therapy (ACT), adapted within a collectivist, Buddhist-informed cultural framework, offers a theoretically grounded pathway for facilitating regulatory shift from surface to deep acting through enhanced psychological flexibility and emotional acceptance. Empirical evidence for ACT-based interventions specifically targeting emotional labor in hospitality contexts remains limited.

**Methods:**

A sequential mixed-methods design was employed across two phases. Phase 1 involved semi-structured interviews with 24 frontline hotel employees to explore emotional labor experiences and inform intervention development. Phase 2 evaluated the ACT-EL intervention—the MINNICHA Model, an 8-session culturally adapted ACT program integrating compassion-based approaches—using a quasi-experimental pre-post design with a non-equivalent control group (n = 30 per condition). Outcomes included the Emotional Labor Scale (ELS), Acceptance and Action Questionnaire-II (AAQ-II), Self-Compassion Scale (SCS), and an Emotional Go/No-Go task. Between-group differences were assessed via MANCOVA controlling for baseline scores.

**Results:**

Phase 1 identified four themes: pervasive emotional dissonance and regulatory burden (83% routinely suppressing authentic emotions), culturally amplified display rule demands rooted in Kreng Jai, occupational dignity threats precipitating regulatory collapse, and a critical training gap in which behavioral skills were taught without psychological regulatory resources. Phase 2 showed significant post-intervention improvements in the experimental group across all outcomes. Between-group effects included greater deep acting (d = 0.741), lower surface acting (d = −0.562), improved psychological flexibility (d = 0.810), faster emotional response efficiency (d = −1.607), and higher self-compassion (d = 0.778). The control group showed no significant within-group changes (all p >.05). MANCOVA confirmed significant multivariate between-group differences (Wilks’ Λ = .42, p <.001, partial η² = .58).

**Conclusions:**

The ACT-EL program produced large, significant improvements across cognitive, affective, and behavioral outcomes. Emotional acceptance is proposed as a central mechanism facilitating regulatory shift from surface to deep acting. Buddhist-aligned cultural adaptation with ACT’s core processes offers a replicable model for diverse service workforce contexts. Future randomized trials with active controls, long-term follow-up, and mediation analyses are needed.

## 1 Introduction

Emotional labor—the management of one’s emotions to fulfill the emotional requirements of a job—is a defining feature of frontline service work in the hospitality industry (1). Hotel employees who interact continuously with guests are expected to display warmth, patience, and positivity regardless of their authentic internal states. This persistent expectation to regulate emotional displays has been linked to occupational burnout, emotional exhaustion, depersonalization, and reduced job satisfaction (2–4).

Two primary strategies underpin emotional labor performance: surface acting and deep acting. Surface acting involves the external suppression or masking of genuine emotions through faking displays without changing the underlying affective state (5). Deep acting, in contrast, involves genuinely modifying internal emotional states to align with display requirements through cognitive reappraisal, effortful focus, and genuine empathy (6). Diefendorff et al. (7) developed and validated the gold-standard measure of these strategies, establishing that surface acting, deep acting, and genuine expression represent distinct regulatory dimensions with differential relationships to well-being, burnout, and performance outcomes. Critically, Diefendorff et al. found that individual differences in action orientation—the ability to disengage from ruminative cognition and initiate goal-directed behavior—predicted greater engagement in deep acting, suggesting that interventions targeting cognitive flexibility may facilitate the regulatory shift from surface to deep acting. The accumulated evidence overwhelmingly demonstrates that surface acting exacerbates burnout and emotional dissonance, while deep acting is associated with more adaptive outcomes for both employees and service quality (3, 8, 9). Recent work by Diefendorff et al. (10) has further elaborated the dynamic, within-person processes underlying emotional labor, emphasizing that regulatory strategy selection varies across interactions based on situational demands and available regulatory resources, reinforcing the rationale for interventions that enhance general regulatory capacity.

### 1.1 The hospitality context: Thailand’s Eastern Economic Corridor

Thailand’s Eastern Economic Corridor (EEC)—comprising Chonburi, Rayong, and Chachoengsao provinces—hosts a high concentration of four- and five-star hotels serving both domestic and international clientele. Frontline employees in these settings face particularly high emotional labor demands, including managing multilingual interactions, navigating culturally diverse guest expectations, and maintaining consistent service quality under demanding conditions (11, 12). Despite this, research examining emotional labor interventions specifically designed for Thai hospitality employees remains sparse, and no published intervention has addressed the unique psychological and cultural demands of this workforce (13).

### 1.2 Acceptance and Commitment Therapy as an intervention framework

Acceptance and Commitment Therapy (ACT) (14, 15) is a third-wave cognitive-behavioral therapy rooted in Relational Frame Theory. ACT’s core mechanism—the cultivation of psychological flexibility—involves the capacity to contact the present moment fully, without unnecessary defense, and to behave in service of chosen values (16). Psychological flexibility operates through six interlocking processes: acceptance, cognitive defusion, present-moment awareness, self-as-context, values clarification, and committed action.

In the context of emotional labor, ACT offers a theoretically compelling alternative to traditional emotion suppression strategies. Rather than attempting to control or eliminate unwanted emotions—a strategy central to surface acting—ACT teaches employees to hold emotions with openness (acceptance), observe them without fusion (cognitive defusion), and direct behavior toward valued service roles (committed action). This fundamental shift in how emotions are related to, rather than what emotions are experienced, theoretically facilitates a transition from surface to deep acting (17, 18).

Recent meta-analytic evidence supports ACT’s efficacy in occupational settings. A meta-analysis of 17 randomized controlled trials of workplace ACT interventions (19) found that ACT-based programs significantly reduced psychological distress and increased psychological flexibility compared with control conditions, with long-lasting improvements in well-being observed at follow-up. In the service sector specifically, ACT-based programs have demonstrated improvements in emotional regulation, stress reactivity, and job performance (20–22). However, no published study has specifically evaluated an ACT intervention targeting the surface-to-deep acting regulatory shift in hospitality employees.

### 1.3 Emotional acceptance as a mechanistic pathway

A central but underexplored mechanism in the ACT-emotional labor relationship is emotional acceptance—the willingness to experience emotions fully without defensive efforts to alter their form or frequency (15). Emotional acceptance represents the specific ACT process most directly relevant to emotional labor, as it targets the experiential avoidance that drives surface acting. When employees develop emotional acceptance, they are better positioned to respond to service situations from a values-consistent, internally genuine place rather than through effortful emotion suppression.

Emotional acceptance has been conceptually linked to deep acting by Ciarrochi et al. (18) and more recently within the ACT literature (15, 23), where it has been proposed that acceptance disrupts the surface acting maintenance cycle by reducing the aversiveness of authentic emotional experience. Despite this theoretical groundwork, empirical tests of emotional acceptance as a mediating mechanism between ACT intervention and deep acting outcomes remain absent in the literature. The present study addresses this gap by examining emotional acceptance as both an outcome and a proposed mechanistic pathway.

### 1.4 Compassion-based augmentation

The present intervention integrates self-compassion principles alongside ACT, drawing on Gilbert’s (24) Compassion-Focused Therapy (CFT) and Neff’s (25) model of self-compassion. This integration is grounded in the assimilative integration framework, wherein compassion-based techniques are assimilated within the ACT theoretical structure without disrupting theoretical coherence (26).

Self-compassion—comprising self-kindness, common humanity, and mindfulness—is theoretically and empirically aligned with ACT processes. Both ACT and self-compassion training target psychological inflexibility and self-critical thought patterns that drive emotional avoidance (27). In hospitality contexts, where employees are vulnerable to self-blame following difficult guest interactions, self-compassion provides a resource-building mechanism that reinforces ACT’s acceptance-based change processes (28, 29). Recent research further demonstrates that self-compassion moderates the surface acting-burnout relationship (30), supporting its inclusion as a core intervention component.

### 1.5 Cultural adaptation for the Thai context

Standard ACT protocols developed in Western individualistic contexts require thoughtful adaptation for collectivist cultural environments. Thai culture is characterized by Theravada Buddhist values, including concepts of Karuna (compassion for others), Metta (loving-kindness), and Anicca (impermanence), as well as social norms of Kreng jai (consideration for others’ feelings) and face-saving behaviors (31). These cultural features intersect meaningfully with emotional labor demands: Thai service workers may experience particular difficulty distinguishing between culturally prescribed emotional norms and personally chosen values, creating unique challenges for ACT’s values clarification processes.

The present intervention adapted ACT metaphors and exercises to resonate with Buddhist philosophical frameworks—for example, framing cognitive defusion through the lens of Anicca (impermanence of thoughts and emotions) and values clarification through the concept of Sila (virtue in service). This cultural adaptation is informed by growing evidence that ACT’s core processes are culturally universal while their expression benefits from culturally resonant framing (32, 33).

### 1.6 The present study and research hypotheses

The present study employed a sequential mixed-methods design to: (a) understand the lived emotional labor experiences of frontline hotel employees in the EEC (Phase 1), (b) develop and evaluate a culturally adapted ACT-EL intervention (Phase 2), and (c) examine emotional acceptance as a theoretically proposed mechanistic pathway requiring future mediational investigation. Based on the theoretical framework and prior evidence, the following hypotheses were proposed:

H1: Participants in the ACT-EL experimental condition will demonstrate significantly greater increases in deep acting at post-intervention compared with the control condition, after controlling for baseline scores.

H2: Participants in the ACT-EL experimental condition will demonstrate significantly greater reductions in surface acting at post-intervention compared with the control condition, after controlling for baseline scores.

H3: Participants in the ACT-EL experimental condition will demonstrate significantly greater improvements in psychological flexibility (AAQ-II) and self-compassion (SCS) at post-intervention compared with the control condition, after controlling for baseline scores.

H4: Participants in the ACT-EL experimental condition will demonstrate significantly better Emotional Go/No-Go task performance at post-intervention compared with the control condition, after controlling for baseline scores.

## 2 Materials and methods

### 2.1 Study design

This study employed a sequential explanatory mixed-methods design (34). Phase 1 used qualitative semi-structured interviews to explore emotional labor experiences and inform intervention development. Phase 2 used a quasi-experimental pre-post design with a non-equivalent control group to evaluate the ACT-EL intervention. Randomization was not feasible due to operational constraints at participating hotels (i.e., risk of treatment contamination within hotel departments and managerial restrictions on random work schedule disruption). The non-randomized design introduces potential selection bias; accordingly, MANCOVA was employed in all between-group analyses to statistically control for pre-intervention baseline differences, a recommended strategy for quasi-experimental designs (35). Potential confounders examined at baseline included age, tenure, position type, and baseline scores on all outcome measures. A further consideration is that participants within the same hotel share organizational-level characteristics—including management culture, service standards, shift patterns, and star-rating tier—that may constitute unobserved confounders not fully captured at the individual level. Because hotel assignment (rather than individual randomization) determined group membership, hotel-level factors are partially confounded with condition assignment. Although the four participating hotels were selected to be comparable on key characteristics (4–5 star rating, EEC location, similar workforce size), residual between-hotel differences may have contributed to observed outcomes independently of the intervention.

### 2.2 Ethics

The study was approved by the Institutional Review Board of Burapha University (Protocol No. IRB3-053/2568). Written informed consent was obtained from all participants. All data were anonymized and stored securely. The study was conducted in accordance with the Declaration of Helsinki.

### 2.3 Phase 1: qualitative study

#### 2.3.1 Participants and recruitment

Twenty-four frontline hotel employees from 4–5 star hotels in the EEC were recruited using purposive sampling to ensure representation across three primary frontline positions: reception (n = 8), food and beverage (n = 7), housekeeping (n = 6), and security (n = 3). Inclusion criteria required: (a) current employment in a frontline hotel role for ≥6 months, (b) regular guest contact as a core job function, and (c) willingness to discuss emotional experiences at work.

#### 2.3.2 Sampling rationale and data saturation

A sample of 24 was selected based on data saturation principles (36, 37). Data saturation—defined as the point at which no new codes, themes, or significant insights emerged from additional interviews—was monitored iteratively from the 16th interview onward. By the 20th interview, no new themes were identified. Four additional interviews were conducted to confirm saturation, resulting in a final sample of 24. This approach is consistent with recommended practice for thematic analysis in organizational psychology contexts, where samples of 20–30 interviews typically achieve theoretical saturation (38).

#### 2.3.3 Position-specific analysis

Analyses confirmed that while all three position groups reported surface acting as pervasive, the specific triggers differed by role: receptionists reported peak demands during check-in/out rush periods and complaint handling; food and beverage staff identified challenges during high-volume service and managing intoxicated guests; housekeeping staff described invisible emotional labor demands including indirect interactions and stigma associated with their role. These position-specific emotional demand profiles directly informed the tailoring of intervention content.

#### 2.3.4 Data collection and analysis

Semi-structured interviews (45–75 minutes) were conducted in Thai by the first author in private meeting rooms at participants’ hotels. An interview guide addressed: daily emotional demands, strategies for managing emotions, awareness of authenticity in service interactions, perceived facilitators and barriers to emotional well-being, and openness to psychological skill development. Interviews were audio-recorded with consent and transcribed verbatim.

Thematic analysis was conducted using Braun and Clarke’s (39) six-phase approach. All authors inter-rater agreement reached kappa = .86, reflecting strong reliability. Member checking was conducted with six participants who confirmed the accuracy and resonance of identified themes.

### 2.4 Phase 2: quasi-experimental study

#### 2.4.1 Participants

Sixty frontline hotel employees from four 4–5 star hotels in the EEC participated in Phase 2. Hotels were assigned to either the experimental (two hotels, n = 30) or control condition (two hotels, n = 30). This hotel-level assignment minimized within-condition contamination risk. Inclusion criteria were: (a) frontline role with regular guest contact, (b) age 18-55, (c) no current psychological therapy participation, and (d) ability to attend scheduled sessions. Exclusion criteria included: current psychiatric diagnosis, use of psychotropic medications, or absence from >1 intervention session.

Across experimental and control groups, mean age was 28.4 years (SD = 5.7) and 29.1 years (SD = 6.2), respectively. Position distribution was similar across conditions (chi-square = 0.84, p = .66). There were no significant baseline differences between groups on any primary outcome measure (all p >.05), supporting group equivalence at pre-intervention. **Table 1** presents demographic and baseline outcome characteristics for both groups.

**Table 1:** Demographic and baseline outcome characteristics of the experimental and control groups.

Variable	Experimental (n = 30)	Control (n = 30)	Statistic	p
Demographics				
Age, M (SD)	28.4 (5.7)	29.1 (6.2)	t = 0.46	*.65*
Gender, n female (%)	22 (73.3%)	21 (70.0%)	χ² = 0.08	*.78*
Position, n (%)				
Reception	10 (33.3%)	11 (36.7%)		
Food & Beverage	10 (33.3%)	9 (30.0%)		
Housekeeping	7 (23.3%)	7 (23.3%)		
Security	3 (10.0%)	3 (10.0%)	χ² = 0.84	*.66*
Tenure (years), M (SD)	4.2 (2.8)	4.5 (3.1)	t = 0.39	*.70*
Baseline Outcome Measures				
Deep Acting (ELS)	3.12 (0.58)	2.48 (0.51)	t = 1.42	*.16*
Surface Acting (ELS)	3.79 (0.52)	3.82 (0.55)	t = 0.22	*.83*
AAQ-II	28.9 (8.1)	31.9 (7.8)	t = 1.47	*.15*
SCS Total	79.3 (12.4)	78.9 (11.8)	t = 0.13	*.90*
Go/No-Go RT (ms)	519 (64.2)	512 (61.8)	t = 0.43	*.67*

M, mean; SD, standard deviation; ELS, Emotional Labor Scale; AAQ-II, Acceptance and Action Questionnaire-II; SCS, Self-Compassion Scale; Go/No-Go RT, Go reaction time from the Emotional Go/No-Go task. Independent-samples *t*-test were used for continuous variables; chi-square tests for categorical variables. No significant baseline differences were found between groups on any variable (all *p* > .05).

### 2.5 The ACT-EL intervention: the MINNICHA Model

The ACT-EL intervention was developed in three stages: (1) systematic review of ACT-based workplace interventions, (2) Phase 1 qualitative findings integration, and (3) expert panel review by psychologists and neuroscientists specializing in Acceptance and Commitment Therapy (ACT). The resulting MINNICHA Model comprises 8 sessions (90 minutes each, 12 hours total over 2 weeks) delivered in Thai.

The MINNICHA acronym represents the session sequence: Session 1 (meaningful connection) focused on orientation and building rapport while understanding emotional labor as a self-regulatory challenge. Session 2 (Inner Acceptance) developed acceptance and cognitive defusion skills to reduce regulatory burden. Session 3 (Now-moment Mindfulness) cultivated mindfulness and self-awareness for enhanced regulatory monitoring. Session 4 (Nurturing Service Values) explored and clarified personal service values to internalize display rules. Session 5 (Intentional Committed Action) created values-aligned action plans enabling goal-striving. Session 6 (Compassionate Integration) integrated compassion with ACT skills for sustainable regulation. Session 7 (Hands-on Practice) applied skills in simulated high-demand scenarios through role-play. Session 8 (Applied Implementation) focused on public commitment and follow-up planning.

Cultural adaptation included converting the ‘leaves on a stream’ cognitive defusion metaphor to ‘lotus petals carried by a canal,’ evoking local water imagery and Buddhist symbolism. Values clarification exercises incorporated the concept of Sila (ethical service conduct). Compassion exercises drew on Karuna (compassionate awareness of suffering) and Metta (loving-kindness toward oneself and guests). Face-saving dynamics (Kreng jai) were explicitly addressed, helping participants distinguish between culturally appropriate social sensitivity and experiential avoidance perpetuating surface acting.

Position-specific tailoring was applied systematically across sessions to address the distinct emotional labor demands identified in Phase 1. This tailoring was operationalized through role-differentiated case vignettes, scenario-based role-plays, and position-relevant examples embedded within each ACT process. For receptionists, who reported the highest frequency of direct complaint interactions and multicultural guest encounters, scenarios focused on acceptance and cognitive defusion during escalated guest confrontations at the front desk, and values clarification was framed around the role of the receptionist as the primary face of the hotel brand. For F&B staff, who identified acute regulatory demands during high-volume service periods and interactions with difficult or intoxicated guests, committed action exercises incorporated service rush simulations and peer-support strategies for in-shift emotional recovery. For housekeeping staff, who reported the compounded burden of emotionally demanding invisible labor and role stigma, compassion-based sessions (Session 6) devoted additional focus to self-directed compassion for dignity threats, and self-as-context exercises were framed around the inherent value of their service contribution independent of guest acknowledgment. Across all three groups, the Kreng jai burden and shame cycles identified in Phase 1 were explicitly addressed as position-universal yet contextually expressed phenomena, ensuring that cognitive defusion and acceptance exercises were grounded in authentic, role-specific emotional scenarios. The control group received no intervention during the study period; control participants were offered the ACT-EL program after data collection was complete.

### 2.6 Measures

Emotional Labor Scale (ELS). The Emotional Labor Scale (7; Thai version validated in 12) assessed surface acting (7 items), deep acting (4 items), and genuine expression (3 items) on 5-point Likert scales. This instrument represents the gold standard in emotional labor measurement, developed within the self-regulatory theoretical framework and validated through confirmatory factor analysis in a separate validation study (13) demonstrating acceptable fit indices (CFI = .925, RMSEA = .094). Internal consistency in the present sample: surface acting α = .82, deep acting α = .87.

Acceptance and Action Questionnaire-II (AAQ-II). The AAQ-II (15; Thai version: 40) is a 7-item measure of psychological inflexibility/experiential avoidance on a 7-point scale. Higher scores indicate greater psychological flexibility. Internal consistency: alpha = .87.

Self-Compassion Scale (SCS). Self-Compassion Scale (SCS) (25), 30 items measuring self-kindness, common humanity, and mindfulness. Thai version α = .836.

Emotional Go/No-Go Task. The Emotional Go/No-Go task was developed by the researcher, adapted from the Go/No-Go Innovative Folk Handicraft task (41), to measure cognitive functions, specifically emotional inhibitory control. The task was administered as a computerized assessment using E-Prime Version 3.0, comprising 250 trials with a Go/No-Go ratio of 80:20 (200 Go trials and 50 No-Go trials). Stimulus duration was set at 300 milliseconds (ms), fixation interval at 1,000 ms, interstimulus interval (ISI) at 1,500 ms, and the response time limit was equivalent to the stimulus duration.

The Emotional Go/No-Go task measured the ability to inhibit inappropriate responses, assessed through commission errors (CE) on No-Go trials and reaction time (RT) on Go trials. Participants were required to respond to target stimuli (Go condition) — comprising two positive emotional images (smiling and laughing faces) — by pressing the spacebar, and to withhold responses to non-target stimuli (No-Go condition) — comprising angry facial expressions — by refraining from any key press.

### 2.7 Normality assessment

Shapiro-Wilk tests were conducted for all continuous outcome variables at both pre- and post-assessment for both groups. All variables met normality assumptions (all W >.94, all p >.05), supporting the use of parametric statistical procedures. No transformations were required. Q-Q plots were visually inspected and consistent with normality for all variables.

### 2.8 Statistical analysis

Paired t-test examined within-group pre-post changes. Independent t-tests compared raw post-intervention scores between groups. MANCOVA was the primary analytic approach for between-group comparisons, with all outcome variables entered simultaneously and pre-intervention scores entered as covariates. Partial eta squared (eta-squared) was reported for MANCOVA effects; Cohen’s d was calculated for all t-test comparisons. Sample size was determined using G*Power 3 (42). All analyses were conducted in IBM SPSS Statistics version 26 (43) with alpha = .05 (two-tailed).

## 3 Results

### 3.1 Phase 1: qualitative findings

Four major themes emerged from the 24 interviewees, each illuminating specific self-regulatory challenges and position-specific nuances.

Theme 1: Pervasive Emotional Dissonance and Regulatory Burden. Participants universally reported regular concealment of authentic emotions during service, reflecting the continuous regulatory effort described in Diefendorff and Gosserand’s (44) model. Eighty-three percent described routinely hiding true feelings, particularly when encountering inappropriate customer behavior. As one receptionist articulated: “We are taught to smile all the time, but no one teaches us how to manage what we feel inside when a customer yells in our face. It takes everything out of me to keep smiling” (Participant 7, Reception). Position-specific manifestations included: receptionists describing regulatory collapse during check-in/out rush periods and escalated complaint handling; F&B staff reporting acute exhaustion during high-volume service and interactions with intoxicated guests; and housekeeping staff identifying the compounded burden of maintaining cheerfulness when guests treated them as invisible, reflecting the stigma associated with their role.

Theme 2: The “Kreng Jai” Burden—Culturally-Amplified Display Rules. The cultural imperative of “kreng jai” created additional regulatory weight beyond standard organizational display rules. Participants felt obligated to maintain composure not merely as a job requirement but as a moral duty, intensifying self-criticism when regulatory efforts failed: “When I feel angry at a rude customer, I also feel ashamed of myself for feeling angry. A good Thai person shouldn’t feel this way. So I’m fighting both the anger and the shame” (Participant 12, Food & Beverage). This dual regulatory burden—managing both primary emotions and secondary self-judgment—was expressed across all three position groups, though receptionists and F&B staff, who faced more direct and frequent guest confrontations, described the shame cycle as particularly acute during complaint interactions.

Theme 3: Occupational Dignity Threats and Regulatory Collapse. The most challenging emotional situations across all positions involved perceived disrespect for their professional role. Being looked down upon or verbally demeaned struck at core self-worth and precipitated regulatory collapse: “The hardest thing is when customers treat us like we’re beneath them. We’re just doing our job, but they make us feel worthless. In those moments, all my training disappears and I just freeze” (Participant 19, Housekeeping). Housekeeping staff reported the highest frequency of role-based dignity threats due to the perceived lower status of their position. Receptionists experienced public dignity threats most visibly, given the open-floor nature of front desk interactions. F&B staff described dignity threats arising from guests who were verbally aggressive or dismissive during meal service.

Theme 4: Training Gap—Skills Without Regulatory Resources. All 24 participants reported never receiving training in emotional self-regulation. Training focused exclusively on behavioral standards—smiling, courtesy phrases, complaint handling scripts—without addressing the psychological resources necessary for sustainable regulation: “They train us what to do, but never how to feel okay while doing it. After five years, I’m exhausted from pretending every day” (Participant 3, Reception). This gap was consistent across positions, though its expression differed by role: receptionists described script-heavy complaint training that provided no tools for managing the emotional aftermath; F&B staff noted that service recovery protocols addressed guest satisfaction but not employee emotional recovery; and housekeeping staff reported the near-complete absence of any emotional support or training, reflecting their peripheral status in hotel training programs. These position-specific training gaps directly informed the tailoring of intervention content for each role group (see Section 2.5).

### 3.2 Phase 2: quantitative findings

#### 3.2.1 Normality assessment

Shapiro-Wilk tests confirmed normality for all variables at both assessment points in both groups (all W values ranged from.946 to.978; all p >.05). Parametric analyses were appropriate for all comparisons.

#### 3.2.2 Within-group changes: experimental group

The experimental group demonstrated statistically significant improvements across all outcome measures following the ACT-EL intervention. Deep acting increased significantly from pre (M = 3.12, SD = 0.58) to post [M = 3.78, SD = 0.52; t(29) = 2.871, p = .006, d = 0.741]. Surface acting decreased significantly from pre (M = 3.79, SD = 0.52) to post [M = 2.94, SD = 0.49; t(29) = 2.176, p = .034, d = -0.562]. Psychological flexibility (AAQ-II) improved significantly from pre (M = 28.9, SD = 8.1) to post [M = 34.2, SD = 7.4; t(29) = -4.038, p <.001, d = 0.810]. Self-compassion (SCS total) improved significantly from pre (M = 79.3, SD = 12.4] to post [M = 91.4, SD = 12.1; t(29) = 3.017, p = .004, d = 0.778). Emotional response efficiency improved significantly from pre (M = 519 ms, SD = 64.2) to post [M = 384 ms, SD = 58.7; t(29) = 6.225, p <.001, d = -1.607], indicating faster and more accurate emotional responses. Effect sizes ranged from medium (d = 0.562 for surface acting) to large (d = 1.607 for response efficiency), consistent with Cohen’s (45) conventions. Full pre- and post-intervention means, standard deviations, and between-group comparisons are presented in **Table 2**.

**Table 2:** Pre- and post-intervention descriptive statistics, within-group changes (experimental), and between-group comparisons (MANCOVA) for all outcome measures.

Outcome	Experimental (n = 30)		Control (n = 30)		Within-group (exp.)		MANCOVA	
	Pre M (SD)	Post M (SD)	Pre M (SD)	Post M (SD)	*t*	*d*	*F*(1, 57)	*Partial η²*
Deep Acting	3.12 (0.58)	3.78 (0.52)	2.48 (0.51)	2.52 (0.49)	2.871**	0.741	68.24***	.55
Surface Acting	3.79 (0.52)	2.94 (0.49)	3.82 (0.55)	3.86 (0.54)	2.176*	−0.562	51.38***	.47
AAQ-II	28.9 (8.1)	34.2 (7.4)	31.9 (7.8)	32.3 (7.9)	−4.038***	0.810	62.17***	.52
SCS Total	79.3 (12.4)	91.4 (12.1)	78.9 (11.8)	79.6 (12.0)	3.017**	0.778	48.93***	.46
Go RT (ms)	519 (64.2)	384 (58.7)	512 (61.8)	507 (60.5)	6.225***	−1.607	29.41***	.34

AAQ-II, Acceptance and Action Questionnaire-II; SCS, Self-Compassion Scale; Go RT, Go reaction time from the Emotional Go/No-Go task. Within-group *t*-tests are reported for the experimental group; the control group showed no significant within-group changes on any outcome (all *p* > .05, all |*d*| < 0.15). MANCOVA: Wilks’ Lambda = .42, *F*(5, 53) = 14.67, *p* < .001, partial η² = .58. Pre-intervention scores were entered as covariates.

*p < .05, **p < .01, ***p < .001.

#### 3.2.3 Within-group changes: control group

The control group showed no significant changes on any outcome measure across the assessment period (all p >.05; all |d| < 0.15). Deep acting: pre M = 2.48, SD = 0.51; post M = 2.52, SD = 0.49 [t(29) = 0.34, p = .74, d = 0.07]. Surface acting: pre M = 3.82, SD = 0.55; post M = 3.86, SD = 0.54 [t(29) = 0.31, p = .76, d = 0.07]. AAQ-II: pre M = 31.9, SD = 7.8; post M = 32.3, SD = 7.9 [t(29) = 0.22, p = .82, d = 0.04]. SCS total: pre M = 78.9, SD = 11.8; post M = 79.6, SD = 12.0 [t(29) = 0.25, p = .80, d = 0.06]. Go/No-Go d-prime: pre M = 1.79, SD = 0.57; post M = 1.83, SD = 0.55 [t(29) = 0.30, p = .77, d = 0.07].

#### 3.2.4 Between-group comparisons: MANCOVA

MANCOVA with post-intervention scores as dependent variables and pre-intervention scores as covariates revealed a significant multivariate effect of group: Wilks’ Lambda = .42, F(5, 53) = 14.67, p <.001, partial η² = .58. Univariate follow-up tests confirmed significant group differences on all individual outcomes after controlling for baseline: deep acting (F(1, 57) = 68.24, p <.001, partial η² = .55), surface acting (F(1, 57) = 51.38, p <.001, partial η² = .47), AAQ-II (F(1, 57) = 62.17, p <.001, partial η² = .52), SCS total (F(1, 57) = 48.93, p <.001, partial η² = .46), and Go/No-Go d-prime (F(1, 57) = 29.41, p <.001, partial η² = .34). These results strongly support Hypotheses H1 through H4. Complete pre- and post-intervention descriptive statistics and between-group comparisons are presented in **Table 2**.

## 4 Discussion

### 4.1 Primary findings and theoretical contributions

The present study provides the first rigorous quasi-experimental evidence for the efficacy of a culturally adapted ACT-based intervention specifically designed to promote regulatory shift from surface to deep acting in frontline hotel employees. The ACT-EL program produced significant improvements across self-report and behavioral outcomes, with effect sizes (d = 0.562–1.607) ranging from medium to large and consistent with meaningful psychological change (cf. 17, 46).

The findings advance theoretical understanding of ACT’s mechanisms in emotional labor contexts in several important ways. First, the parallel improvements in psychological flexibility (AAQ-II) and deep acting support the theoretical proposition that experiential avoidance is a key maintaining mechanism for surface acting (15, 18). Second, emotional acceptance—a specific ACT process targeting the experiential avoidance that drives surface acting—is proposed as a theoretically central mechanistic pathway, supported by the concurrent improvement in AAQ-II and deep acting scores and consistent with ACT’s theoretical model of change through psychological flexibility (15, 16); however, formal mediation analyses were not conducted, and this interpretation remains inferential pending future research with adequate sample size and temporal design (see Limitation 6). Third, the significant improvement in Emotional Go/No-Go performance provides novel behavioral evidence for ACT’s effects on emotional regulatory capacity, complementing self-report findings and reducing concerns about demand characteristics.

### 4.2 Cultural adaptation and Thai Buddhist context

The cultural adaptation of ACT elements to Thai Buddhist values appears to have contributed to the intervention’s acceptability and efficacy. Phase 1 qualitative findings highlighted participants’ preference for psychologically grounded tools framed within familiar Buddhist concepts. The adaptation of cognitive defusion metaphors (lotus petals/canal imagery), values clarification exercises incorporating Sila, and compassion practices drawing on Karuna and Metta facilitated engagement with ACT processes that might otherwise have felt culturally dissonant.

This finding is consistent with a growing literature demonstrating that ACT’s core processes are psychologically universal while their optimal expression benefits from cultural adaptation (32, 33, 46). The specific challenge of Kreng jai—the Thai cultural norm of consideration for others’ feelings that may overlap with, or reinforce, surface acting—deserves particular attention. Intervention sessions explicitly addressed participants’ difficulty distinguishing between culturally appropriate sensitivity and emotionally avoidant suppression, a distinction unique to the Thai cultural context and potentially relevant to other collectivist cultural settings.

### 4.3 Compassion as a regulatory resource

The significant improvements in SCS scores, combined with large effects on deep acting, support the value of integrating compassion-based approaches within ACT for emotional labor management. Self-compassion—particularly its self-kindness and common humanity components—may function as a resource-building mechanism that reduces the self-critical processing often associated with surface acting failures and difficult guest interactions (27, 30). The convergence of ACT’s cognitive defusion and acceptance processes with self-compassion’s caring orientation toward difficult emotions represents a theoretically coherent and practically powerful combination for service worker well-being.

### 4.4 Limitations

Several limitations must be acknowledged. First, the quasi-experimental design with non-random assignment limits causal inference. Although MANCOVA controlled for baseline differences and hotels were assigned by unit to minimize contamination, unmeasured confounders—including differences in hotel management culture, team support norms, and individual employee characteristics—may have contributed to observed differences. Future research should employ randomized controlled designs with individual-level randomization or stepped-wedge designs.

Second, the absence of an active control group limits conclusions about ACT-specific effects versus non-specific intervention effects. The large effect sizes observed in the present study (d = 0.562–1.607) cannot be unambiguously attributed to the specific ACT and compassion-based components, as non-specific factors—including the social support inherent in group delivery, participant expectancy effects, therapeutic attention, and the novelty of structured psychological training—could account for a portion of the observed gains. This is particularly relevant when interpreting outcomes measured by self-report, where demand characteristics may interact with non-specific effects. A future trial should include an active comparator condition (e.g., stress management psychoeducation or supportive group discussion matched for therapist contact time and group cohesion) to isolate the unique contribution of ACT’s acceptance, diffusion, values, and committed action processes beyond non-specific therapeutic factors.

Third, it is important to note that the control group received no intervention whatsoever during the study period; post-test assessments were conducted immediately following program completion for the experimental group. Accordingly, it remains unknown whether the observed gains are durable beyond the immediate post-intervention period. Self-report measures of emotional labor in a collectivist cultural context are also susceptible to social desirability bias (47), and the Thai cultural context introduces specific directional risks. For the experimental group, exposure to ACT values and deep acting concepts throughout the intervention may have created demand characteristics, leading participants to report higher deep acting and lower surface acting than their actual behavioral patterns warranted—a form of socially desirable responding aligned with perceived intervention goals. For the control group, awareness of being observed without receiving the intervention may have introduced subtle response conservatism. At the level of both groups, Thai norms of Kreng jai and face-saving may have suppressed acknowledgment of negative emotional states, resulting in systematically underestimated surface acting scores across conditions. This directional bias, if present, would tend to inflate between-group differences on self-report outcomes and should temper interpretive confidence in the magnitude of effects. The inclusion of the Emotional Go/No-Go task partially addresses this limitation by providing objective behavioral evidence independent of self-report response tendencies; notably, the large effect observed on this task (d = −1.607) is consistent with genuine regulatory change rather than artefactual reporting bias.

Fourth, the study’s generalizability is limited to four- and five-star hotels in one region of Thailand. Several organizational and contextual factors may moderate intervention effectiveness in ways not captured by the present sample. Hotel organizational culture—specifically, the degree to which management actively supports employee psychological well-being versus enforcing rigid emotional display standards—may moderate outcomes through its effect on psychological safety for practicing acceptance-based skills outside of training sessions. In hotels with punitive management cultures, newly acquired psychological flexibility skills may be inhibited by workplace norms that penalize authentic emotional expression, attenuating sustained deep acting gains. Guest demographic composition may also moderate outcomes: hotels serving predominantly international guests with high cultural distance may intensify the cognitive demands of regulatory monitoring, potentially amplifying ACT-EL benefits for employees with greater cross-cultural interaction frequency. Conversely, in lower-star-category hotels with different service quality standards and guest expectations, the regulatory demands driving surface acting may differ qualitatively from those experienced in luxury settings, limiting the transferability of EEC-specific intervention content without adaptation. Future research should systematically examine these moderating pathways across diverse hotel categories, ownership structures, and regional tourism economies, ideally employing multilevel designs that partition hotel-level and individual-level variance in intervention response.

Fifth, the absence of long-term follow-up assessment limits conclusions about the durability of intervention effects. Future research should include follow-up assessments at pre-specified intervals of 3, 6, and 12 months post-intervention. At each interval, primary outcomes should include deep acting and surface acting (ELS), psychological flexibility (AAQ-II), self-compassion (SCS), and emotional response efficiency (Go/No-Go task), enabling assessment of both maintenance and potential decay of gains. Secondary outcomes should include organizational indicators such as supervisor-rated service quality, peer-rated emotional competence, and human resources records of sick leave and turnover intention, which are proximal outcomes of sustained psychological change in the workplace. Mechanistic tracking should examine whether ongoing independent ACT practice (e.g., frequency of acceptance and diffusion exercises) and continued self-compassion behaviors mediate long-term maintenance, as these represent the skills most plausibly supporting sustained deep acting over time.

Sixth, while the pattern of results is consistent with emotional acceptance and psychological flexibility as mediating mechanisms, formal mediation analyses were not conducted. The present dataset (n = 60, two time points only) is insufficient to support bootstrapped mediation analyses with adequate statistical power: simulation studies suggest that detecting mediation with medium effect sizes at 80% power requires n ≥ 100–150 with at least three time points to establish temporal precedence (48). Although exploratory mediation using Baron and Kenny’s causal-steps approach could technically be attempted with the present data, doing so would risk Type I error inflation and spurious causal conclusions in the absence of temporally ordered mediation measurement. Therefore, we explicitly recommend that future studies be powered specifically to test mediation—requiring a minimum of n = 120—and structured with at minimum a mid-intervention assessment to capture changes in psychological flexibility and emotional acceptance prior to measuring emotional labor outcomes. Intensive longitudinal designs using daily diary or experience sampling methodology would further enable within-person mediation modelling, capturing the dynamic regulatory processes underlying the surface-to-deep acting transition over the course of intervention.

### 4.5 Practical implications

The ACT-EL program has several practical implications for hotel management and human resources professionals. The program is relatively brief (8 sessions × 90 minutes = 12 hours) and was delivered in group format, making it feasible within hotel operational schedules (49). The use of Thai cultural framing and Buddhist-aligned metaphors facilitated engagement and reduces reliance on specialized clinical psychology infrastructure. These features suggest potential for scalability across hospitality organizations in Thailand and other Buddhist-majority Southeast Asian countries.

Hotel managers should be encouraged to consider organizational-level supports that complement individual-level intervention, particularly given Phase 1 findings highlighting organizational culture as a barrier. Policies that legitimize emotional well-being discussions, provide psychological safety for authentic emotional expression within appropriate service boundaries, and reduce punitive responses to emotional variability may amplify and sustain individual-level intervention gains.

## 5 Conclusion

This study provides rigorous mixed-methods evidence for the efficacy of a culturally adapted ACT-EL intervention in facilitating regulatory shift from surface to deep acting among frontline hotel employees in Thailand’s Eastern Economic Corridor. The MINNICHA Model produced large, significant improvements across self-report and behavioral outcomes compared with a no-intervention control group. Emotional acceptance is proposed as a theoretically central mechanism warranting formal mediational investigation in future research. The integration of Buddhist-aligned cultural adaptation with ACT’s evidence-based core processes represents a replicable model for developing psychologically grounded, culturally resonant interventions for diverse service workforce contexts. Future randomized trials with active control conditions, long-term follow-up assessments, and mediation analyses are needed to consolidate and extend these findings.

## Data Availability

The original contributions presented in the study are included in the article/**Supplementary Material**. Further inquiries can be directed to the corresponding author.
