# Supplementary material for: Beyond surface acting: a mixed-methods investigation of an ACT-based intervention for promoting psychological flexibility and regulatory shift in hotel frontline emotional labor
Source: Front Psychiatry. 2026 May 25;17:1785171. doi: 10.3389/fpsyt.2026.1785171 (PMC13243413; doi:10.3389/fpsyt.2026.1785171)
Supplement: Supplementary file 1 [file DataSheet1.pdf]

IN-DEPTH INTERVIEW GUIDE

Emotional Labor Experiences of Frontline Hotel Service Employees

Thailand's Eastern Economic Corridor (EEC)

Supplementary Material S1

Interview Information

Interview Code: ________________ Date: ________________ Time: ________________

Location: ________________ Duration: ________________ minutes

Interviewer: ________________

PART 1: DEMOGRAPHIC INFORMATION

Instructions: Please mark (✓) in the box that best describes you.

1. Gender

☐ Male

☐ Female

☐ Prefer not to say

2. Age

☐ Under 25 years

☐ 25 – 35 years

☐ 36 – 45 years

☐ 46 – 55 years

☐ Over 55 years

3. Education Level

☐ Below Bachelor's degree (High school / Vocational certificate / Diploma)

☐ Bachelor's degree

☐ Master's degree

☐ Doctoral degree

4. Work Experience in Frontline Service Position

☐ Less than 1 year

☐ 1 – 5 years

☐ 5 – 10 years

☐ More than 10 years

5. Current Department / Position

☐ Front Office / Reception

☐ Food & Beverage Service

☐ Housekeeping

☐ Concierge / Guest Relations

☐ Security

☐ Other: ________________

6. Hotel Star Rating

☐ 4-star hotel

☐ 5-star hotel

PART 2: IN-DEPTH INTERVIEW QUESTIONS

Instructions: These questions serve as a guide for semi-structured interviews. Interviewers may adapt the sequence and follow up with probing questions based on participant responses. Estimated duration: 45-60 minutes.

Domain 1: Experience as a Frontline Service Employee

Question 1.1: Please describe your work experience as a frontline service employee. How long have you been working in this role, and what are your main responsibilities?

- *Probe: What does a typical workday look like for you?*
- *Probe: How many customers do you typically interact with per day?*

Response:

Question 1.2: On a daily basis, how do you interact and communicate with customers? What is the nature of these interactions?

- *Probe: Can you describe the range of customer types you encounter?*
- *Probe: What proportion of interactions would you describe as positive vs. challenging?*

Response:

Question 1.3: What inspired you to choose a career in frontline hospitality service? What motivates you to continue in this role?

- *Probe: Has your motivation changed over time?*

Response:

Domain 2: Challenges and Emotional Difficulties in Frontline Service Work

Question 2.1: From your experience, please describe the most common problems or challenges you encounter while serving customers.

- *Probe: Can you share a specific example of a particularly difficult situation?*
- *Probe: How frequently do you encounter such situations?*

Response:

Question 2.2: When facing stressful situations or dealing with angry, demanding, or difficult customers, how do you feel internally? How do you manage those feelings?

- *Probe: What physical sensations do you notice in your body during these moments?*
- *Probe: What thoughts typically go through your mind?*
- *Probe: Do you feel you need to hide or suppress your true feelings? If so, how?*

Response:

Question 2.3: What is the biggest challenge you face in maintaining appropriate emotional expressions while providing service?

- *Probe: Are there times when you feel like you're "acting" or "putting on a mask"?*
- *Probe: How does this affect you after your shift ends?*

Response:

Question 2.4: In Thai culture, we have the concept of "kreng jai" (being considerate of others' feelings). How does this cultural value influence how you handle your emotions at work?

- *Probe: Do you feel additional pressure to maintain composure because of cultural expectations?*
- *Probe: How do you balance being "kreng jai" with your own emotional needs?*

Response:

Domain 3: Self-Reflection and Self-Regulatory Strategies

Question 3.1: In challenging situations, what do you think about or tell yourself before speaking or acting that helps you manage your emotions effectively?

- *Probe: Do you have any mental strategies or self-talk techniques?*
- *Probe: Where did you learn these strategies?*

Response:

Question 3.2: How do you think your ability to manage emotions during challenging situations affects the quality of service you provide?

- *Probe: Can you give an example where emotional management led to a better outcome?*
- *Probe: What about a time when it didn't go well?*

Response:

Question 3.3: What aspects of your work as a frontline service employee are you most proud of?

- *Probe: What personal qualities help you succeed in this role?*

Response:

Question 3.4: After particularly difficult interactions, how do you recover emotionally? What helps you "reset"?

- *Probe: Do you receive any support from colleagues or supervisors?*
- *Probe: How long does it typically take you to recover?*

Response:

Domain 4: Training Needs and Organizational Support

Question 4.1: What kind of organizational support do you think would help you manage your emotions better while working?

- *Probe: What support currently exists? What is missing?*
- *Probe: What would ideal support look like?*

Response:

Question 4.2: Have you ever received any training specifically focused on emotional management or stress management? If yes, please describe. If no, what training do you feel you need?

- *Probe: What topics would be most helpful to learn about?*
- *Probe: What format would work best for you (workshops, online, coaching)?*

Response:

Question 4.3: In the future, what changes or improvements would you like to see in terms of support for emotional well-being among frontline service employees?

- *Probe: What would make the biggest difference in your daily work life?*

Response:

Domain 5: Recommendations for Developing Frontline Service Employees

Question 5.1: Based on your experience, what methods, techniques, or skills do you think would be most effective for helping frontline employees manage their emotions at work?

- *Probe: What has worked for you personally?*
- *Probe: What have you seen work for colleagues?*

Response:

Question 5.2: If a training program were developed specifically to help frontline employees manage emotional challenges at work, what should it include? How should it be delivered?

- *Probe: How long should such training be?*
- *Probe: Should it be during work hours or outside?*
- *Probe: Group or individual format?*

Response:

Question 5.3: Is there anything else you would like to share about your experience with emotional challenges in frontline service work that we haven't discussed?

Response:

INTERVIEW CLOSING

Thank you very much for taking the time to share your experiences and insights with us today. Your input is invaluable for understanding the emotional labor challenges faced by frontline hotel employees and will help us develop effective support programs.

Before we conclude:

- ☐ Do you have any questions for me?
- ☐ Would you be interested in learning about the results of this study?
- ☐ May we contact you for follow-up questions if needed?

Contact Information for Follow-up (optional): ________________________________

INTERVIEWER NOTES

Post-Interview Observations:

Participant's emotional state during interview:
____

Key themes observed: ____

Non-verbal cues noted: ____

Data quality assessment: ____

Additional comments: ____

ETHICAL REMINDER

- Ensure informed consent was obtained before interview
- Remind participant of confidentiality and anonymity protections
- Store all recordings and notes securely according to IRB protocol
- Ethics Approval: Human Research Ethics Committee, Burapha University (IRB3-053/2568)
