# Supplementary material for: Beyond surface acting: a mixed-methods investigation of an ACT-based intervention for promoting psychological flexibility and regulatory shift in hotel frontline emotional labor
Source: Front Psychiatry. 2026 May 25;17:1785171. doi: 10.3389/fpsyt.2026.1785171 (PMC13243413; doi:10.3389/fpsyt.2026.1785171)
Supplement: Supplementary file 2 [file DataSheet2.pdf]

SUPPLEMENTARY MATERIAL S2

Full Statistical Outputs: Within-Group Pre-Post Comparisons

Table S1. Within-Group Pre-Post Comparison for the Experimental Group (n = 30)

Paired-samples t-test results comparing pre-test and post-test scores following participation in the ACT-EL program

Measure	Time Point	M	SD	MD	t	p	Cohen's d
Emotional Labor							
Surface Acting	Pre-test	17.30	6.45	-3.43	3.017	.002**	-0.636
	Post-test	13.87	4.09				
Deep Acting	Pre-test	11.60	3.23	2.47	-4.174	<.001***	0.715
	Post-test	14.07	3.88				
Emotional Go/No-Go Task							
Go Accuracy (%)	Pre-test	98.32	5.71	0.18	0.581	.565	0.108
	Post-test	98.50	7.10				
Go RT (ms)	Pre-test	401.39	46.61	-38.83	4.546	<.001***	-1.013
	Post-test	362.57	32.10				
Commission Errors (%)	Pre-test	5.07	4.96	-1.30	1.427	.164	-0.265
	Post-test	3.77	3.74				
Self-Compassion							
Compassion	Pre-test	17.40	2.30	-0.63	1.597	.121	-0.296
	Post-test	16.77	2.93				
Psychological Flexibility							
AAQ-II Score	Pre-test	38.00	8.34	3.07	-4.038	<.001***	0.750
	Post-test	41.07	6.51				

*Note. M = Mean; SD = Standard Deviation; MD = Mean Difference (Post - Pre); df = 29 for all comparisons. *p < .05, **p < .01, ***p < .001. Effect sizes interpreted as: small (d = 0.20), medium (d = 0.50), large (d = 0.80).*

Results Narrative: Experimental Group Pre-Post Changes

Emotional Labor. Paired-samples t-tests revealed significant changes in emotional labor strategies following participation in the ACT-EL program. Surface acting scores decreased significantly from pre-test (*M* = 17.30, *SD* = 6.45) to post-test (*M* = 13.87, *SD* = 4.09), *t*(29) = 3.017, *p* = .002, with a medium effect size (*d* = -0.636). Conversely, deep acting scores increased significantly from pre-test (*M* = 11.60, *SD* = 3.23) to post-test (*M* = 14.07, *SD* = 3.88), *t*(29) = -4.174, *p* < .001, with a medium-to-large effect

size (d = 0.715). These findings indicate a successful regulatory shift from response-focused (surface acting) to antecedent-focused (deep acting) emotional labor strategies.

Emotional Inhibitory Control. Analysis of the Emotional Go/No-Go task revealed differential patterns across indicators. Go accuracy showed no significant change from pre-test (M = 98.32%, SD = 5.71) to post-test (M = 98.50%, SD = 7.10), p = .565, likely due to ceiling effects. However, Go reaction time (RT) decreased significantly from pre-test (M = 401.39 ms, SD = 46.61) to post-test (M = 362.57 ms, SD = 32.10), $t(29)$ = 4.546, $p < .001$, with a large effect size (d = -1.013), indicating enhanced efficiency in emotional response processing. Commission errors showed a non-significant reduction from 5.07% to 3.77% (p = .164).

Compassion. Self-compassion scores showed a non-significant decrease from pre-test (M = 17.40, SD = 2.30) to post-test (M = 16.77, SD = 2.93), $t(29)$ = 1.597, p = .121, d = -0.296.

Psychological Flexibility. Psychological flexibility, measured by the AAQ-II, increased significantly from pre-test (M = 38.00, SD = 8.34) to post-test (M = 41.07, SD = 6.51), $t(29)$ = -4.038, $p < .001$, with a medium-to-large effect size (d = 0.750). This finding supports the theoretical proposition that psychological flexibility serves as the core mechanism of change in ACT-based interventions.

Table S2. Within-Group Pre-Post Comparison for the Control Group (n = 30)

Paired-samples t-test results comparing pre-test and post-test scores for the waitlist control group

Measure	Time Point	M	SD	MD	t	p	Cohen's d
Emotional Labor							
Surface Acting	Pre-test	17.10	7.89	0.33	-0.385	.703	0.072
	Post-test	17.43	7.99				
Deep Acting	Pre-test	10.03	3.98	1.00	-1.393	.173	0.259
	Post-test	11.03	4.30				
Emotional Go/No-Go Task							
Go Accuracy (%)	Pre-test	98.27	2.41	-1.55	0.796	.432	-0.148
	Post-test	96.72	10.91				
Go RT (ms)	Pre-test	424.66	43.15	-2.25	0.303	.764	-0.056
	Post-test	422.40	41.73				
Commission Errors (%)	Pre-test	5.53	4.42	-2.00	2.673	.012*	-0.497
	Post-test	3.53	4.43				
Self-Compassion							
Compassion	Pre-test	16.57	2.87	-0.23	0.559	.580	-0.104
	Post-test	16.34	2.83				
Psychological Flexibility							
AAQ-II Score	Pre-test	42.07	5.33	-0.37	0.410	.685	-0.076
	Post-test	41.70	6.51				

*Note. M = Mean; SD = Standard Deviation; MD = Mean Difference (Post - Pre); df = 29 for all comparisons. *p < .05, **p < .01, ***p < .001. Effect sizes interpreted as: small (d = 0.20), medium (d = 0.50), large (d = 0.80).*

Results Narrative: Control Group Pre-Post Changes

Emotional Labor. The control group showed no significant changes in emotional labor strategies over the study period. Surface acting scores remained stable from pre-test (*M* = 17.10, *SD* = 7.89) to post-test (*M* = 17.43, *SD* = 7.99), *t*(29) = -0.385, *p* = .703, *d* = 0.072. Similarly, deep acting scores showed no significant change from pre-test (*M* = 10.03, *SD* = 3.98) to post-test (*M* = 11.03, *SD* = 4.30), *t*(29) = -1.393, *p* = .173, *d* = 0.259. These null findings rule out maturation or testing effects as alternative explanations for changes observed in the experimental group.

Emotional Inhibitory Control. Go accuracy showed no significant change from pre-test (*M* = 98.27%, *SD* = 2.41) to post-test (*M* = 96.72%, *SD* = 10.91), *p* = .432. Go reaction time remained essentially unchanged from pre-test (*M* = 424.66 ms, *SD* = 43.15) to post-test (*M* = 422.40 ms, *SD* = 41.73), *p* = .764,

d = -0.056. Notably, commission errors decreased significantly from pre-test (*M* = 5.53%, *SD* = 4.42) to post-test (*M* = 3.53%, *SD* = 4.43), *t*(29) = 2.673, *p* = .012, *d* = -0.497, which may reflect practice effects from repeated testing.

Compassion. Self-compassion scores remained stable from pre-test (*M* = 16.57, *SD* = 2.87) to post-test (*M* = 16.34, *SD* = 2.83), *t*(29) = 0.559, *p* = .580, *d* = -0.104.

Psychological Flexibility. Psychological flexibility remained unchanged from pre-test (*M* = 42.07, *SD* = 5.33) to post-test (*M* = 41.70, *SD* = 6.51), *t*(29) = 0.410, *p* = .685, *d* = -0.076. The stability of psychological flexibility in the control group, contrasted with significant improvement in the experimental group, provides evidence that changes in the experimental group were attributable to the ACT-EL intervention rather than extraneous factors.

Table S3. Summary Comparison of Effect Sizes Between Groups

Measure	Experimental Group Cohen's d	Control Group Cohen's d	Interpretation
Surface Acting	-0.636**	0.072	Intervention effect confirmed
Deep Acting	0.715***	0.259	Intervention effect confirmed
Go Accuracy (%)	0.108	-0.148	Ceiling effect; no change expected
Go RT (ms)	-1.013***	-0.056	Intervention effect confirmed
Commission Errors (%)	-0.265	-0.497*	Practice effect in both groups
Compassion	-0.296	-0.104	No significant change
Psychological Flexibility	0.750***	-0.076	Intervention effect confirmed

Note. *$p < .05$, **$p < .01$, ***$p < .001$. *Effect size benchmarks: small ($d = 0.20$), medium ($d = 0.50$), large ($d = 0.80$).*

Summary of Key Findings

The within-group analyses provide strong evidence for the effectiveness of the ACT-EL program. The experimental group demonstrated significant improvements in four key outcomes following the intervention: (1) decreased surface acting (medium effect), (2) increased deep acting (medium-to-large effect), (3) faster emotional response times (large effect), and (4) enhanced psychological flexibility (medium-to-large effect). In contrast, the control group showed no significant changes on these measures, ruling out maturation, testing effects, or regression to the mean as alternative explanations. The pattern of results supports the theoretical proposition that ACT-based interventions facilitate regulatory shift by enhancing psychological flexibility, which in turn enables more adaptive emotional labor strategies.
